# A neural disconnection hypothesis on impaired numerical processing

**DOI:** 10.3389/fnhum.2013.00663

**Published:** 2013-10-22

**Authors:** Elise Klein, Korbinian Moeller, Klaus Willmes

**Affiliations:** ^1^Knowledge Media Research Center, Tuebingen, Germany; ^2^Department of Neurology, Section Neuropsychology, University Hospital, RWTH Aachen University, Aachen, Germany

**Keywords:** numerical cognition, mental arithmetic, processing pathways, arithmetic fact retrieval, disconnection syndromes

In recent years the number of fMRI studies aiming to pinpoint the neural substrates of number processing increased steadily (e.g., Dehaene et al., 2003; see Dehaene, 2009; Nieder and Dehaene, 2009 for reviews; see Arsalidou and Taylor, 2010 for a meta-analysis). Nevertheless, while considerable progress has been made, the neural correlates and cognitive mechanisms involved in the retrieval of arithmetic facts such as for multiplication (e.g., 2 × 3 = 6) or for addition (e.g., 2 + 3 = 5) fact knowledge is still a matter of debate. According to the currently most influential model of numerical cognition, the Triple Code Model by Dehaene and colleagues (Dehaene and Cohen, 1995, 1997; Dehaene et al., 2003) the retrieval of arithmetic fact knowledge is verbally mediated and subserved by a neural system relying on left-hemispheric perisylvian language areas and the angular gyrus. It is thus assumed that arithmetic facts are retrieved directly from long-term memory without the need of any magnitude manipulations. Instead, the verbally mediated representation format is claimed to trigger the retrieval of a word sequence from memory, which is associated with the AG (e.g., “two times three equals six”).

Consistent with this prediction of the Triple Code Model, evidence from neuroimaging (e.g., Rickard et al., 2000; Delazer et al., 2003, 2005; Ischebeck et al., 2006, 2007; Grabner et al., 2009; Zamarian et al., 2009) as well as single-case studies with brain damaged patients (e.g., Hittmair-Delazer et al., 1994; Lee, 2000; Cohen et al., 2000) point to an involvement of the left angular gyrus (AG) in the retrieval of arithmetic facts. Compelling evidence for a functional involvement of the left AG comes from a training study by Delazer et al. (2003). In their study adults were trained on complex multiplication problems (e.g., 17 × 26 =) over 5 days. In a subsequent fMRI session, contrasts between trained and untrained problems revealed stronger activation in the left angular gyrus for the trained than the untrained set interpreted to indicate increased reliance on fact-retrieval to solve trained problems. In contrast, higher activation in frontal cortices and in the lPS for the untrained problems was suggested to reflect higher demands on executive functions and magnitude manipulations, respectively. In sum, these activation differences, in particular between the IPS and the angular gyrus for trained vs. untrained problems indicated a transition from quantity-based processing to direct fact retrieval (for similar results, see Ischebeck et al., 2007).

However, there is also contradictory evidence in the literature. For instance, Van Harskamp et al. (2005) reported a patient with a severe multiplication fact-retrieval deficit, although his brain lesion did not involve the left AG. More particularly, a detailed review of single-case studies of patients, for whom neuroradiological images are available, revealed that also the lesion of patient BOO (Dehaene and Cohen, 1997), who presented with a selective impairment for multiplication, did not involve the AG, the supramarginal gyrus (SMG) or perisylvian language areas. This can be seen from the axial lesion drawings in Figure 3 (p. 226). Moreover, in patient ATH who also exhibited a severe impairment for rote multiplication (Cohen and Dehaene, 2000), the posterior part of the angular gyrus seemed spared. Additionally, Van Harskamp and Cipolotti (2001) reported a patient VP who presented with a selective impairment for simple multiplication problems and extended atrophy due to Alzheimer's disease, while the left angular gyrus was relatively spared. Finally, Zaunmueller et al. (2009) reported a patient with a severe multiplication fact-retrieval deficit although his brain lesion did neither involve the left AG or even left-hemispheric language areas.

Thus, at a first glance these results do not seem to be in concordance with the notion that the left AG is critical for the retrieval of arithmetic (multiplication) facts nor that this structure in conjunction with temporal, frontal and subcortical areas is indeed critical for arithmetic (multiplication) fact knowledge as suggested by the Triple Code Model.

## An alternative account to explain the seemingly contradictory evidence

In the present opinion paper we wish to point out that taking into account fiber pathways of the brain may offer an alternative explanation for the symptoms observed without necessarily contradicting the propositions of the Triple Code Model. Employing probabilistic fiber tracking, Klein et al. (2013) recently suggested that magnitude- and fact retrieval-related processing are subserved by two anatomically largely distinct fronto-parietal networks, both of them comprising dorsal and ventral connections. Importantly, for the case of arithmetic fact retrieval these connections between parietal and frontal cortex encompassed the extreme (EmC) and the external (EC) capsule system for the ventral pathways and the superior longitudinal fascicule (SLF) for the dorsal pathway.

**Figure 1:**
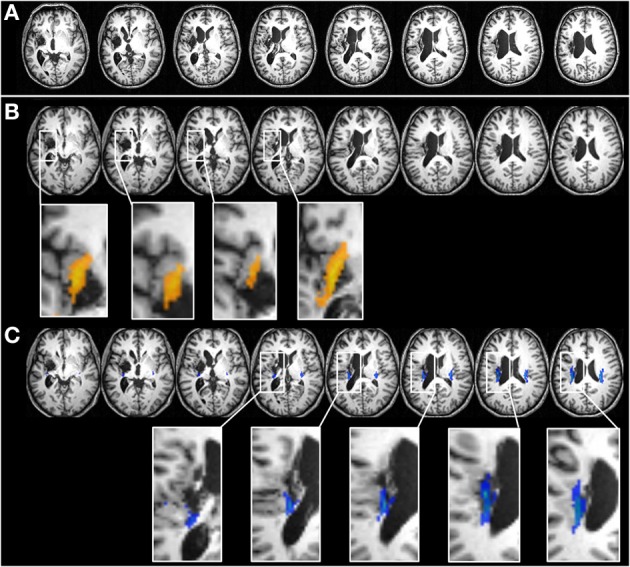
**Detailed views on the lesion of patient WT in axial orientation. (A)** WT's lesion in native space. **(B,C)** WT's lesion transformed into MNI space. In **(B)**, the enlarged squares of specific slices ventral fiber tracts of the EC/EmC system are displayed additionally in orange. As can be seen from these detailed pictures, the ventral pathways connecting angular gyrus/temporal perisylvian language areas with Broca's area in the inferior temporal gyrus (BA 45) are completely distrupted by the lesion. **(C)** Depicts the superior longitudinal fascicule (SLF) in a detailed view on patient WT's brain in axial orientation (given in blue color). Again, as can be seen in the enlarged squares of specific slice, the dorsal SLF system is lesioned as well. Abbreviations: BA, Brodmann area; EC, external capsule; EmC, extreme capsule.

To pursue this idea, we re-analysed the data of a study in our lab on patient WT originally published in 2009 (Zaunmueller et al., 2009). WT was a 49-year-old right-handed former post-office clerk who suffered from a hemorrhage resulting in a perifocal edema affecting his left basal ganglia. MRI scanning 29 months post onset showed a large left-hemisphere lesion involving subcortical as well as cortical regions and an enlarged left lateral ventricle. The lesion affected the insula, putamen, pallidum, parts of the thalamus, and the internal capsule (see Figures 1A,B). Importantly, the lesion also comprised parts of the inferior and superior longitudinal fasciculus (SLF) as well as the EmC and the EC. To investigate our proposition of disconnected white matter tracts more closely, the T1-weighted anatomical scan, performed on a 3.0 T Philips Achieva scanner using a standard head coil (*TE* = 4.6 ms, *TR* = 9.9 ms, voxel size = 1 × 1 × 1 mm^3^, *FOV* = 256 mm, matrix = 256 × 256, 180 slices, 1 mm thickness), was normalized into standard stereotaxic MNI (Montreal Neurological Institute) coordinates space using SPM8 [http://www.fil.ion.ucl.ac.uk/spm]. White matter tracts were identified according to the DTI-based JuBrain atlas (Bürgel et al., 2006) implemented in the Anatomy Toolbox of the Juelich Research Center for the white matter fiber tracts.

The EC/EmC system is a ventral pathway travelling between insula and putamen connecting the AG to the inferior frontal gyrus, pars triangularis (BA 45, Broca's area; see Klein et al., 2013). The EC/EmC system corresponds to rostral/anterior parts of the bundle termed *inferior occipitofrontal fasciculus* (IFOF/IOF) by other authors (Catani et al., 2002; Suchan et al., 2013). To demonstrate that the EC/EmC system is completely disconnected in patient WT, we added the IOF of the JuBrain atlas to the patient's axial slices (depicted in orange, see Figure 1B). Moreover, inspection of the contralateral (intact) hemisphere also indicates that the EC/EmC system of the left hemisphere is lesioned, because the EmC and EC are easy to detect, separated by the thin layer of the claustrum. In Panel C of Figure 1 the SLF is indicated in blue and inspection of the figure clearly indicates that the dorsal SLF system is disconnected as well. Taken together, we found a disconnection of both the dorsal as well as the ventral fiber pathways connecting the angular gyrus and perisylvian language areas with frontal areas such as Broca's area in patient WT. The lesion of the ventral EC/EmC system as well as the dorsal SLF was reported in the original study but not discussed any further.

While we still agree with the original interpretation that a lesion of the basal ganglia might have induced or at least added to the severe multiplication impairment of WT we wish to suggest that the observed disconnections of both the dorsal and ventral fiber pathway systems may also account for the multiplication impairment of WT. Even though the left AG and the left-hemispheric perisylvian language areas were not affected by his lesion, these areas were no longer connected to frontal areas such as Broca's area as well as the basal ganglia. This interpretation is further corroborated by the fact that training WT extensively daily in two training sessions per day over 4 weeks on multiplication facts not only resulted in a dramatic improvement of multiplication performance but also in a significant increase of activation in the homologue right angular gyrus. Because both dorsal and ventral connections to the left parietal cortex were disrupted, this drill training may not have altered the fMRI signal in the left AG. However, probably encompassing the cingulum, the intact fiber pathway system of the right hemisphere might have led to the situation that arithmetic fact retrieval was possibly taken over by the right hemisphere after extensive training.

We are confident that a similar pathology might account for other so far inconsistent observations in the literature as well. For instance, although white matter lesions were not reported in the study of Van Harskamp et al. (2005), a closer inspection of the MRI scans provided Figure 1, p. 743 and Figure 2, p. 744) suggests lesions of both the ventral EC/EmC system as well as the dorsal SLF. Additionally, the axial illustrations of patient BOO (Dehaene and Cohen, 1997) strongly suggest a complete disconnection of at least the EC/EmC system (Figure 3, p. 226).

Nevertheless, it is important to note that the current conclusions are based on a retrospective analysis of recent single case study, in which some details of the participant's native space scans might have been obstructed during the normalization processes, when reanalyzing the MRI data. Furthermore, it should be considered that all presently available DTI atlases (and the method itself) have their shortcomings. In the present study we chose the JuBrain atlas by Bürgel (Bürgel et al., 2006) instead of DTI based atlases (e.g., Catani and Thiebaut de Schotten, 2012), because the JuBrain atlas also takes into account cytoarchitectonic analyses from post mortem brains, whereas solely DTI-based atlases only provide an indirect measurement of possible fiber tracts. Taken together, we suggest that future prospective studies are highly desirable. For instance, one might think of voxel-based lesion mapping data from a large number of patients to consider not only cortical structures, but also subcortical white matter tracts as well as basal ganglia affections. However, it also needs to be emphasized that the TCM as the most influential model in numerical cognition research was originally based strongly on single-case patient studies as well (already including references to basal ganglia affections, e.g., Dehaene and Cohen, 1995). Therefore, we are confident that the present data are worthwhile, suggesting future researchers to move on from “corticocentric myopia” (Parvizi, 2009) to approaches taking into account the whole architecture of the human brain including non-cortical structures.

In summary, for the interpretation of previous as well as future single-case studies or voxel-based lesion mapping data we strongly recommend to not only investigate gray matter lesions but to also inspect MRI/CT scans for disconnections of white matter fiber pathways and consider the impact of lesions affecting them in the interpretation of the data. In a first next step, it would be interesting to evaluate the idea of disconnected brain areas to account for specific numerical deficits for the case of selectively impaired magnitude processing (as for instance in patients MAR, Dehaene and Cohen, 1997). Interestingly, Klein et al. (2013) observed two anatomically largely distinct networks to underlie arithmetic fact retrieval and number magnitude processing. Therefore, the disconnection hypothesis may even be capable of explaining observed dissociations between fact retrieval and magnitude processing (e.g., MAR and BOO in Dehaene and Cohen, 1997).
